# Effect of captopril on radiation-induced TGF-β1 secretion in EA.Hy926 human umbilical vein endothelial cells

**DOI:** 10.18632/oncotarget.15356

**Published:** 2017-02-15

**Authors:** Jingni Wei, Hui Xu, Yinyin Liu, Baiyu Li, Fuxiang Zhou

**Affiliations:** ^1^ Department of Radiation and Medical Oncology, Zhongnan Hospital, Wuhan University, Wuhan, 430071, China; ^2^ Hubei Key Laboratory of Tumor Biological Behaviors, Zhongnan Hospital, Wuhan University, Wuhan, 430071, China; ^3^ Hubei Clinical Cancer Study Centre, Zhongnan Hospital, Wuhan University, Wuhan, 430071, China; ^4^ Department of Radiation Oncology, Cancer Hospital of Guangxi Medical University, Nanning, Guangxi, 530021, China

**Keywords:** irradiation, EA.Hy926, TGF-β, captopril, NF-κB

## Abstract

The pathophysiological mechanism involved in the sustained endothelial secretion of cytokines that leads to fibrosis 6–16 months after radiotherapy remains unclear. Angiotensin II (Ang II) is produced by the endothelium in response to stressing stimuli, like radiation, and may induce the synthesis of TGF-β, a profibrotic cytokine. In this study we tested the hypothesis that captopril, an angiotensin-converting enzyme (ACE) inhibitor, inhibits or attenuates radiation-induced endothelial TGF-β1 secretion. The human endothelial hybrid cell line EA.HY926 was irradiated with split doses of x-rays (28 Gy delivered in 14 fractions of 2 Gy). TGF-β1 mRNA, TNF-α mRNA and TGF-β1 protein levels were evaluated by RT-PCR and western blotting each month until the fifth month post radiation. Ang II was detected using radioimmunoassays, NF-κB activity was examined using EMSA, and western blotting was used to detect the expression of Iκ-Bα. To explore the role of Ang II on radiation-induced TGF-β1 release and Iκ-Bα expression, captopril was added to cultured cells before, during, or after irradiation. Sustained strong expression of TGF-β1 was observed after conventional fractionated irradiation. TNF-α, Ang II, and NF-κB activity were also increased in EA.Hy926 cells after radiation. Captopril decreased Ang II expression, inhibited the NF-κB pathway and reduced TGF-β1 expression. These data suggest that captopril might protect the endothelium from radiation-induced injury.

## INTRODUCTION

Radiotherapy has shown to increase the overall survival and to relief symptoms in cancer patients. Nevertheless, it usually induces normal tissue injury, including radiation fibrosis, a common complication characterized by excess fibroblast proliferation and collagen fiber deposition [1]. Acute and late tissue damage following irradiation have both been linked to the endothelium irrigating normal tissues. Irradiation leads to endothelial cell apoptosis, increased vascular permeability, and acquisition of a pro-inflammatory phenotype [2]. Cytokines related to radiation-induced injury include transforming growth factor-β (TGF-β), interleukin-1 (IL-1), tumor necrosis factor α (TNF-α), platelet-derived growth factor (PDGF), and interleukin-6 (IL-6), among which the function of TGF-β1 has been extensively appraised [3]. A marked increase in TGF-β1 production by endothelial cells in irradiated mice was observed by Pineda et al. [4]. Early variations of TGF-β1 levels during three-dimensional conformal radiation therapy (3D-CRT) were significantly associated with the risk of radiation pneumonitis [5].

Angiotensin II (Ang II) and aldosterone are integral components of the renin-angiotensin-aldosterone system (RAAS), widely expressed in the heart, blood vessel wall, lung, brain, and other tissues and organs. In both early and late phase results, local Ang II levels increased in rat lungs receiving a 20 Gy radiation dose [6]. The potential pro-inflammatory properties of Ang II have been hypothesized to contribute to radiation-induced organ fibrosis in the heart [7]. In the kidney, Ang II was shown to induce fibrogenesis through NF-κB-dependent upregulation of ICAM-1 [8]. Moreover, the Ang II/superoxide/NFκB signaling pathway was shown to regulate the excitability of aortic baroreceptors during chronic heart failure [9]. Ang II receptor antagonists inhibit various signaling pathways involved in the regulation of inflammation, apoptosis and angiogenesis, both in cancer cells and in normal cells, and have shown effectiveness in the treatment of cancer by inhibiting tumor progression, vascularization and metastasis [10]. The inflammatory reaction to a multiplicity of insults includes the production of various cytokines and chemokines, such as TNF-α, which disrupts the barrier function and decreases the thickness of the corneal epithelium [11]. The expression of the Ang II receptor AT1 is increased in irradiated tissues and contributes, at least in part by activating TGF-β1 expression, to the pathophysiology of radiation-induced lung injury, a fatal condition featuring interstitial pneumonitis and fibrosis [12–14].

However, there are no reports of how Ang II mediates radiation-induced activation of TGF-β1 in endothelial cells. Using the human umbilical vein endothelial cell line EA.Hy926, we established an endothelial cell model to study the mechanism of sustained secretion of TGF-β1 induced by ionizing radiation. We also investigated whether Ang II-mediated activation of NFκB contributed to TGF-β1 secretion in irradiated cells. In addition, the effects of captopril, an angiotensin-converting enzyme (ACE) inhibitor, on Ang II and cytokine secretion were also explored, to provide a new theoretical basis and experimental evidence for the prevention and treatment of late radiation-induced injuries.

## RESULTS

### Radiation exposure induces sustained TGF-β1 secretion in EA.Hy926 cells

**Figure 1:**
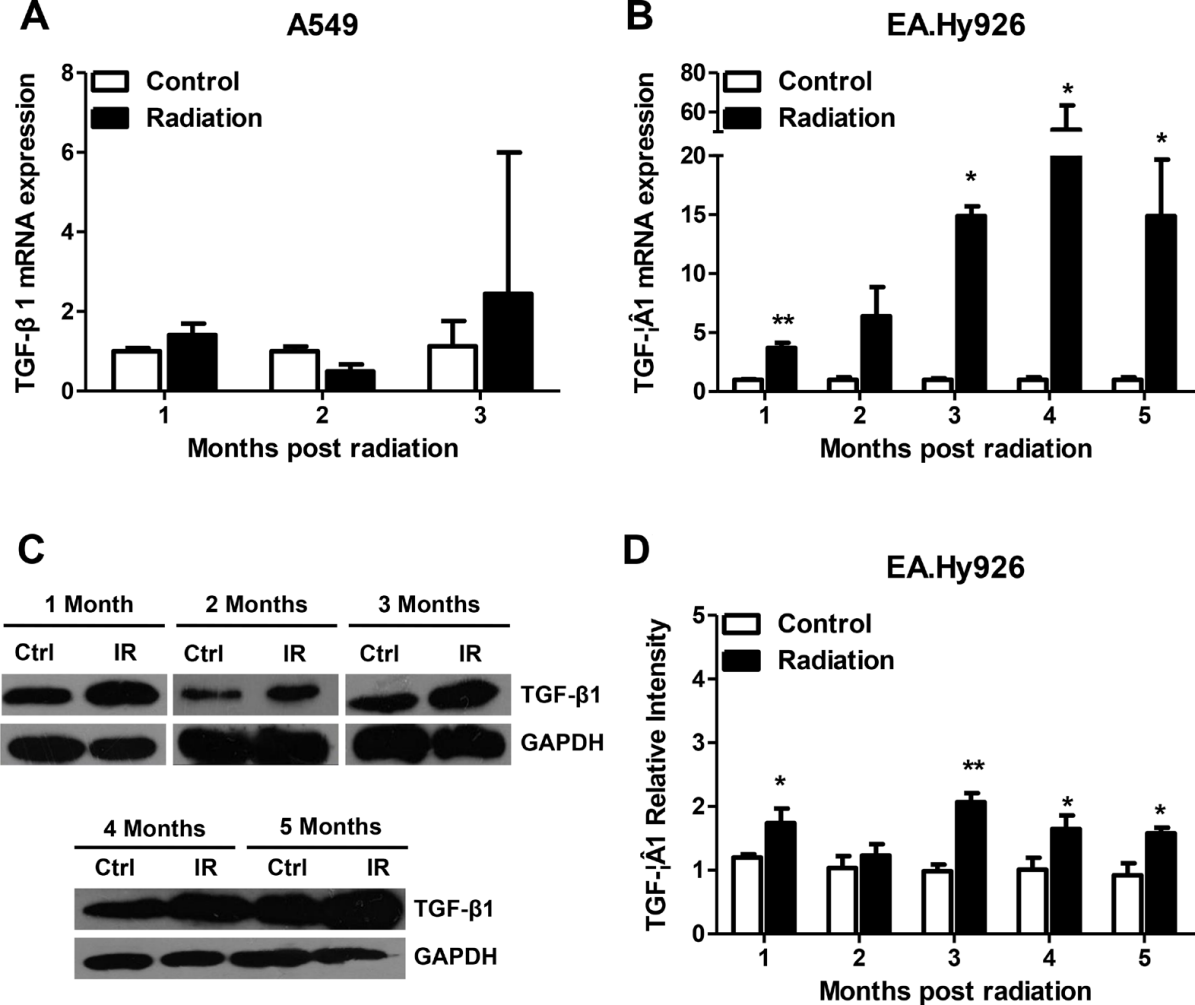
Sustained secretion of TGF-β1 in irradiated EA.Hy926 cells (**A**) After exposure to ionizing radiation, no sustained expression of TGF-β1 mRNA was detected in A549 cells. (**B**) From the first month to the fifth month following 28 Gy radiation, the expression of TGF-β1 mRNA in EA.Hy926 cells increased significantly compared with the control group (*P* < 0.05). (**C** and **D**) TGF-β1 protein levels varied consistently with the mRNA changes (*P* < 0.05). *P < 0.05 compared to the control group; **P < 0.01 compared to the control group.

A cell line model with sustained TGF-β1 secretion was obtained by irradiating human umbilical vein endothelial EA.Hy926 cells. This hybrid cell line, which manifests the biological behavior of endothelial cells, was obtained from the fusion of primary human umbilical vein endothelial cells with A549 human lung adenocarcinoma cells [15]. Therefore, the A549 cell line was used as control in this study. Sustained secretion of the cytokine TGF-β1 in EA.Hy926 cells was achieved after conventional fractionated irradiation (2 Gy/min once a day, five times a week, for a total 28 Gy). The model cell line was named EA.Hy926/TGF-β1. We found that in the process of applying ionizing radiation, radiation-sensitive EA.Hy926 cells showed a longer proliferation cycle and a slower growth rate. In contrast, A549 cells had radiation-resistance characteristics, with no significant changes in cell cycle length or growth rate. After exposure to ionizing radiation, no significant overexpression of TGF-β1 mRNA was detected in A549 cells (Figure 1A). In contrast, from the first to the fifth month after radiation, the expression of TGF-β1 mRNA increased significantly in EA.Hy926 cells (Figure 1B). TGF-β1 protein levels, as assessed by Western blot, were also increased one to five months after irradiation (Figures 1C and 1D).

### Radiation induces TNF-α and Ang II expression

**Figure 2:**
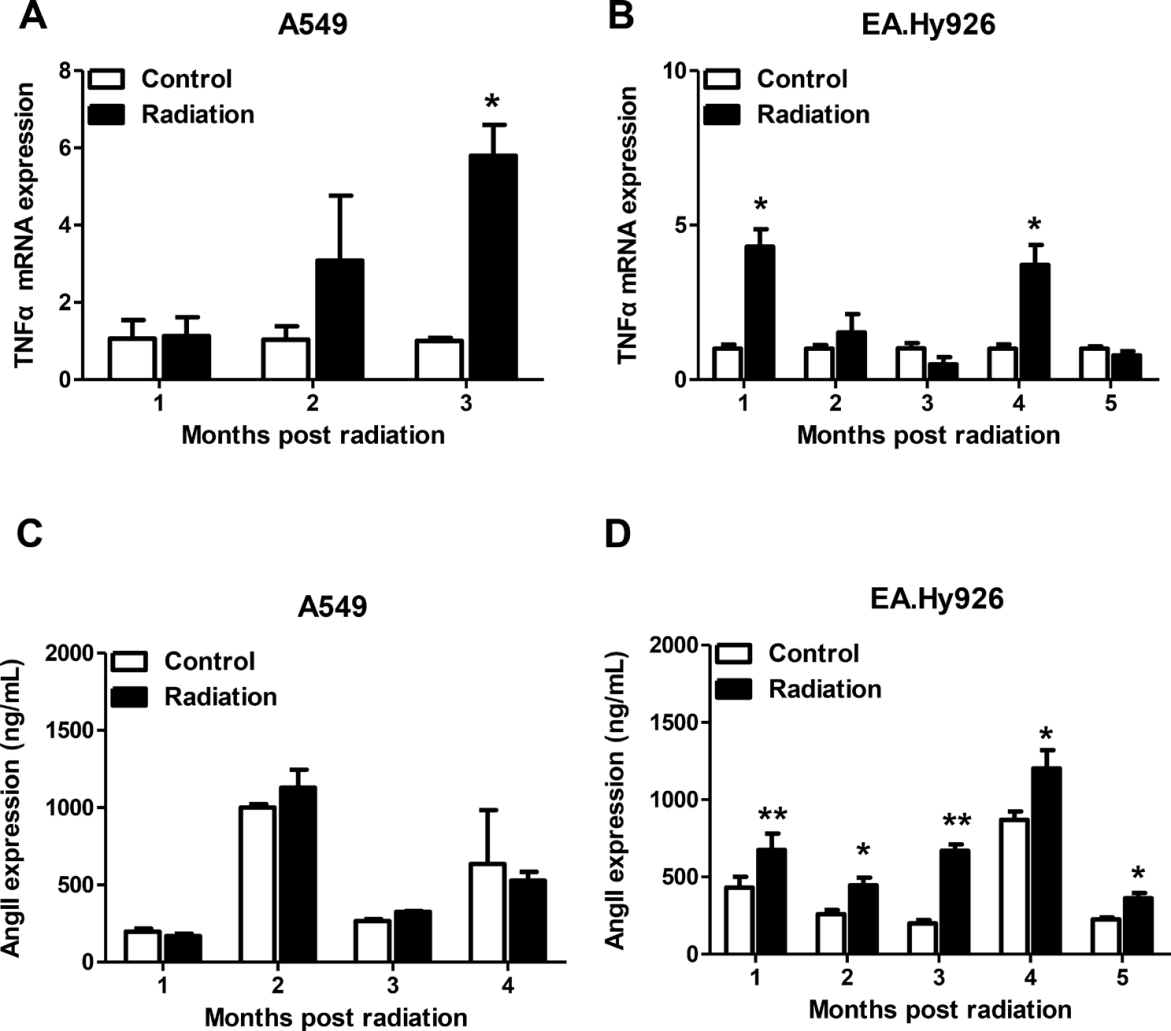
Effects of radiation on TNF-α and Ang II mRNA expression (**A**) Significantly higher TNF-α mRNA expression was observed three months after irradiation in A549 cells. (**B**) TNF-α mRNA levels increased significantly at one and four months after irradiation in EA.Hy926 cells. (**C**) No significant Ang II mRNA expression changes were observed after radiation exposure in A549 cells. (**D**) Compared with control cells, Ang II expression increased significantly in EA.Hy926 cells one to four months after radiation exposure. *P < 0.05 compared to the control group; **P < 0.01 compared to the control group.

The effect of radiation on TNF-α and Ang II mRNA expression was evaluated by real time PCR in EA.Hy926 and A549 control cells. Whereas a significant increase in the expression of TNF-α mRNA was seen 3 months after radiation in A549 cells (Figure 2A), TNF-α mRNA levels in EA.Hy926 cells increased 4-fold one month after radiation, decreased between months 2 and 3, and increased again at month four post-radiation, compared with non-irradiated cells (Figure 2B). Our *in vitro* results may correlate with those obtained in animal models of radiation-induced lung damage, where TNF-α, primarily overexpressed in pulmonary epithelial cells, likely initiated with other cytokines the recruitment of macrophages and other inflammatory cells to inflammation sites very early post irradiation [16, 17]. Ionizing radiation activates various signaling pathways involved in the inflammatory process. As Ang II is a proinflammatory mediator in normal and tumor cells, we explored its expression in irradiated EA.Hy926 and A549 cells. Whereas no significant changes in Ang II mRNA expression were observed after radiation in A549 cells (Figure 2C), Ang II secretion was significantly increased in irradiated EA.Hy926 cells at all time points (Figure 2D).

### Radiation activates the NF-κB pathway

**Figure 3:**
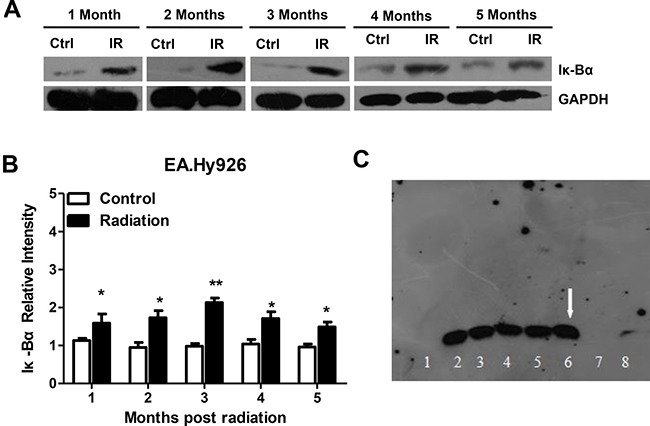
Radiation activates the NF-κB pathway in EA.Hy926 cells (**A** and **B**) Compared with control cells, Iκ-Bα protein levels were significantly elevated after irradiation. (**C**) EMSA assay showing NF-κB activation in irradiated EA.Hy926 cells. Lane 1: EA.Hy926 no radiation Negative control; Lane 2: EA.Hy926 no radiation Experimental group; Lane 3: EA.Hy926 no radiation Cold competition group; Lane 4: EA.Hy926 no radiation Competition mutation group; Lane 5: EA.Hy926 2 Gy × 14 Negative control; Lane 6: EA.Hy926 2 Gy × 14 Experimental group; Lane 7: EA.Hy926 2 Gy × 14 Cold competition group; Lane 8: EA.Hy926 2 Gy × 14 Competition mutation group. *P < 0.05 compared to the control group; **P < 0.01 compared to the control group.

NFκB is a transcription factor that regulates the expression of a number of genes involved in pathophysiological states such as inflammatory disorders. In the resting state, NFκB remains inactive in the cytosol by tightly binding to the specific inhibitor of κBα (IκBα) [18]. In response to multiple stimuli in pathophysiological conditions, IκB molecules are phosphorylated, ubiquitinated and then degraded, thus releasing their inhibition on NFκB [9]. To examine whether NFκB activation status in EA.Hy926 cells is affected by radiation, nuclear proteins were extracted at different time points after the last irradiation and Iκ-Bα protein levels and NF-κB activity were examined by western blotting and EMSA assay, respectively. Compared with non-irradiated, control cells, Iκ-Bα protein levels were significantly elevated from one to five months after radiation (Figure 3A and 3B). EMSA assay showed that NF-κB activity was also activated in EA.Hy926 cells after radiation (Figure 3C).

### Captopril blocks Ang II expression

**Figure 4:**
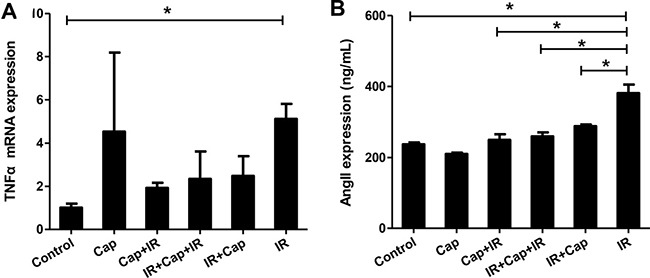
Captopril blocks angiotensin II expression in EA.Hy926 cells (**A**) After the last irradiation, quantitative real-time RT-PCR was used to detect TNF-α. Cap treatment had no effect on radiation-induced TNF-α mRNA levels. (**B**) After the last irradiation, Ang II expression in the cell supernatants was measured by radioimmunoassay. While Cap alone had no effect, Cap treatment before IR, after IR, or in-between IR all inhibited Ang II expression induced by IR. *P < 0.05 compared to the control group; **P < 0.01 compared to the control group.

Many studies in both animal models and humans showed that ACE inhibitors have many beneficial effects on the endothelial function. One such ACE inhibitor, captopril, protected the endothelium against free radical injury in a dose-dependent manner in isolated rabbit abdominal aortas, and this protective effect was related to superoxide anion scavenging [19]. In order to explore the effect of captopril (Cap) on TNF-α expression and Ang II secretion in EA.HY926 cells exposed to ionizing radiation (IR), we established 6 experimental groups: (1) No treatment (Control); (2) Cap only (Cap); (3) Cap first, then IR, 2Gy × 14 times (Cap+IR); (4) IR, 2Gy × 7 times plus Cap, then IR, 2Gy × 7 times (IR+Cap+IR); (5) IR first, 2Gy × 14 times and then Cap (IR+Cap); (6) IR only, 2Gy × 14 times (IR). The maximally effective concentration of Cap for the EA.HY926 cell line was 10^–3^M, as per MTT assay. After the last irradiation, quantitative real-time RT-PCR was used to detect TNF-α mRNA. The results showed that Cap treatment did not affect the increase in TNF-α mRNA elicited by radiation (Figure 4A). In parallel experiments, Ang II was measured by radioimmunoassay in cell culture supernatants. While Cap alone had no effect on baseline Ang II secretion, Cap treatment before IR, after IR, and in-betweenIR sets was, in all cases, able to inhibit radiation-induced Ang II secretion (Figure 4B).

### Captopril reduces radiation-induced TGF-β1 expression by inhibiting the transcriptional activity of NF-κB

**Figure 5:**
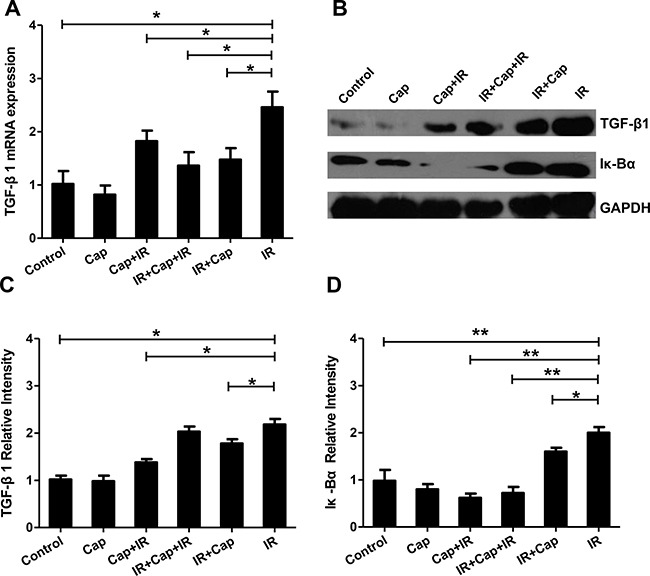
Captopril reduces TGF-β1 expression by inhibiting the transcriptional activity of NF-κB (**A**) Cap treatment before IR, after IR, or in-between IR significantly decreased TGF-β1 mRNA expression induced by IR (**B**). TGF-β1 and Iκ-Bα protein expression assessed by Western blot. After irradiation, TGF-β1 protein changes were coincident with the changes observed in TGF-β1 mRNA. Densitometry analysis showed that while Cap treatment alone did not affect the expression of either protein, it decreased IR-induced TGF-β1 (**C**) and Iκ-Bα (**D**) protein levels.

Ang II inhibitors increase the therapeutic index of radiotherapy by protecting normal cells and sensitizing tumor cells [19]. After irradiation, the expression of TGF-β1 and Iκ-Bα were detected by western bloting in EA.HY926 cells. While Cap alone had no effect on baseline TGF-β1 mRNA expression, its application before IR, after IR, and in-between IR sets inhibited, in all cases, the increase in TGF-β1 expression induced by radiation (Figure 5A). TGF-β1 protein level changes were consistent with the observed mRNA changes (Figure 5B and 5C). Similarly to TGF-β1, Cap treatment before IR, after IR and in-between IR sets inhibited IκBα expression induced by radiation (Figure 5B and 5D). Thus, our data suggests that the ACE inhibitor captopril, applied before, in-between, or after irradiation, decreased TGF-β1 expression by inhibiting Ang II production and NF-κB transcriptional activity. Therefore, captopril may attenuate some adverse effects of TGF-β upregulation during radiotherapy, suchs as facilitation of the epithelial-mesenchymal transition and excessive tissue fibrosis.

## DISCUSSION

This work supports the concept that the endothelium strongly contributes to the progression of radiation-induced fibrosis by releasing TGF-β1. To the best of our knowledge, this is the first study to explore the feasibility of using an ACE inhibitor, captopril, to ameliorate radiation-induced endothelial dysfunction.

Radiotherapy is one of the most effective therapies in cancer treatment. During radiotherapy, ionizing radiation is delivered to cancerous tissue, affecting cells through the production of free radicals and reactive oxygen species (ROS) [20]. However, The activation of a proinflammatory cytokine network has been shown to mediate normal tissue injury in cancer patients treated with radiation [10]. Therefore, side effects caused by radiotherapy are generally unavoidable, and most patients will develop acute complications as well as some degree of chronic damage such as fibrosis [21]. An increasing amount of evidence indicates that endothelial dysfunction plays a critical role in both early and delayed radiation-induced injuries in a variety of normal tissues [22]. Park et al. showed that exposure to radiation induced vascular hyperpermeability in a dose-dependent manner [23]. Induction of vascular hyperpermeability is one of the early vascular responses to radiation exposure and is thought to contribute to subsequent fibrosis and tissue injuries. However, the mechanism underlying radiation-induced hyperpermeability has not yet been clearly elucidated [24].

Ang II is a multifunctional hormone that influences the function of cardiovascular cells through a complex series of intracellular signaling events initiated by its interaction with AT1 and AT2 receptors. AT1 receptor activation leads to cell growth, vascular contraction, inflammatory responses and salt and water retention [25]. In our study, radiation also induced vascular endothelial cells to secrete Ang II, which might activate NF-κB transcription factor activity [25].

Several reports examined the link between radiation and Ang II fluctuations. In rat lungs, the expression of both Ang II and aldosterone increased with increasing radiation doses, and the difference was still observed as time progressed [27]. Ang II binding to AT1 receptors causes superoxide production mainly through activation of NADPH oxidase [25]. In hypertensive Dahl salt-sensitive (DS) rats, impaired endothelium-dependent relaxation to acetylcholine and to insulin is mechanistically linked to upregulation of Ang II actions, production of ROS, and activation of the proinflammatory transcription factor NF-κB [28]. The effects of Ang II on endothelial function have been examined in E-V290M mice infused with a subpressor dose of Ang II or saline for 2 wk. Increased p65 and decreased Iκ-Bα, suggesting increased NF-κB activity, were observed in the aorta from Ang II-infused mice [29].

TGF-β1 has been implicated as a potent stimulator of fibrosis, and could promote the differentiation and proliferation of myofibroblasts to stimulate collagen synthesis [30]. Studies have indicated that in the early events after radiation, serum and lung TGF-β1 levels were increased [31]. Ang II signaling stimulates TGF-β1 and fibronectin production [32]. In contrast, adiponectin has been shown to attenuate Ang II -induced TGF-β1 production in human mesangial cells via an AMPK-dependent pathway [32]. In this study, TGF-β1 levels in irradiated EA.Hy926 cells increased markedly compared with the control group. Moreover, these levels increased over time. These findings may be in good correlate with in *vivo* data showing that treatment with TGF-β antagonists at the time of irradiation significantly reduced acute pneumonitis as well as late-phase fibrosis sixth months after lung irradiation in rats [33], and enhanced the antitumor effect of radiotherapy in a Lewis lung cancer mouse model [34]. Along these lines, another study showed that multimodal therapy that combined pirfenidone, an inhibitor of TGF-induced collagen production, with radiation and sunitinib, significantly reduced tumor growth, as compared to radiation alone [35].

Mixed findings about the effects of ACE inhibitors on radiation-induced injury have been reported. ACE inhibition affects hematopoietic recovery following radiation by modulating the hematopoietic progenitor cell cycle. ACE inhibitors, as well as Ang II receptor blockers such as losartan, inhibited cell growth, decreased c-myc expression and increased apoptosis in leukemia cell lines [36]. We found that the ACE inhibitor captopril blocked radiation-induced Ang II secretion (which would weaken the transcriptional activity of NF-κB), and reduced TGF-β1 mRNA and protein expression. In a clinical report, non-small cell lung cancer (NSCLC) patients taking ACE inhibitors to treat hypertension had better outcome after radiotherapy [37]. Similarly, Kharofa et al. also reported decreased risk of radiation pneumonitis with incidental concurrent use of ACE inhibitors and thoracic radiation therapy [38].

In this study we also examined the expression of TNF-α, another important pro-inflammatory cytokine whose levels are known to be altered by radiation. TNF-α expression increased in the first month after ionizing radiation in EA.Hy926 cells, compared with a late expression surge (after 3 months) observed in A549 control cells. This response suggests that TNF-α might also play a role in early endothelial dysfunction following ionizing radiation.

In summary, we established a human endothelial model cell line, EA.Hy926/ TGF-β1, in which sustained expresion of TGF-β1 was achieved after irradiation. Our model cell line may be useful to study the role of TGF-β1 on endothelial dysfunction induced by radiotherapy. In addition to TGF-β1, radiation prompted the synthesis of the pro-inflammatory cytokine TNF-α, and upregulated Ang II synthesis and NF-κB transcriptional activity. We propose that Ang II overexpression is an early mediator of endothelial dysfunction, by stimulating NF-κB transcriptional activity and inducing the synthesis of proinflammatory cytokines such as TGF-β1, resulting in vascular damage and tissue fibrosis. ACE inhibitors such as captopril might be useful to protect the endothelium and normal tissues against radiation injury.

## MATERIALS AND METHODS

### Cell culture and treatment

The human umbilical vein endothelial cell line EA.Hy926 (GNHu39) and the human non-small cell lung cancer cell line A549 (TCHu150) were purchased from the Cell Resource Center of Shanghai Institutes for Biological Sciences (SIBS) of the Chinese Academy of Sciences. The cells were cultured at 37°C under humidified 5% CO_2_, 95% air in DMEM medium supplied with 10% FBS. To establish the radiation-induced, sustained TGF-β1-secretion model, exponentially growing A549 and EA.Hy926 cells were irradiated by X-ray with a Varian accelerator at 2 Gy split dose (2 Gy/min once a day, five times a week, for a total 28 Gy). Captopril (Invitrogen) 10^–3^ M was applied to cell cultures for 24 hours before, in-between-or after IR. And the lag time between treatment and measurements was 1 week.

### Quantitative real-time PCR

After treatment, total RNA was prepared by using a total RNA Kit (R6934, Omega Bio-tek Inc., GA, U.S.A.) and cDNA was synthesized with a cDNA Synthesis Kit (K1622, Fermentas International Inc., Canada) according to the manufacturer's instructions [39]. Each PCR was performed in triplicate in a final volume of 20 μL solution: 10 μL of SYBR Green dye, 1 μL of diluted cDNA products, 0.2 μM of each paired primer, and 8.6 μL of deionized water. Protocols were as follows: initial denaturation for 5 min at 94°C, followed by 40 cycles of denaturation for 30s at 94°C, and extension for 30s at 58°C. The last cycle for dissociation of SYBR Green probe was 15s at 95°C, 30s at 60°C and 15s at 95°C. The primer sequences for TGF-β1 were: sense, 5′- CAG CAA CAA TTC CTG GCG ATA -3′; antisense, 5′- AAG GCG AAA GCC CTC AAT TT -3′. The primer sequences for TNF-α were: sense, 5′- TCT TCT CGA ACC CCG AGT GA -3′; antisense, 5′- CCT CTG ATG GCA CCA CCA G -3′. The primer sequences for GAPDH were: sense, 5′- CAC CAG GGC TGC TTT TAA CTC TGG TA -3′; antisense, 5′- CCT TGA CGG TGC CAT GGA ATT TGC -3′. Threshold cycle (Ct) values were measured and normalized to that of GAPDH and expressed as a relative ratio.

### Western blot analysis

Total proteins were extracted after treatment using a standard method and the protein concentrations were determined using the Bradford method. The cell lysates were separated by SDS-PAGE and transferred to a PVDF membrane. The membrane with proteins was blocked in TBST containing 5% nonfat milk for 30 min at RT, followed by overnight incubation at 4°C with primary antibodies [1:200; Anti-TGF beta 1 (2Ar2), ab64715, Abcam; anti-TNF alpha (2C8), ab8348, Abcam; anti-IκBα (L35A5), mouse mAb (Amino-terminal Antigen) #4814, Cell Signaling Technology; anti-GAPDH (6C5), ab8245, Abcam]. Blots were washed with TBST, followed by the addition of the secondary antibodies (Goat anti-Mouse IgG (H+L), HRP conjugate; 1:1000; Goat anti-Rabbit IgG (H+L) Superclonal™ Antibody, HRP conjugate, 1:1000; Thermo Fisher Scientific) at 37°C for 1h. Bound antibodies were detected with enhanced chemiluminescence (ECL) reagents (Amersham, Cleveland, OH, USA) and membranes were exposed to Hyperfilm (Amersham, Cleveland, OH, USA). The intensity values of TGF-β1 and GAPDH proteins were measured with Image J software (National Institutes of Health), normalized to that of GAPDH, and expressed as a relative ratio [40].

### Measurement of Ang II levels by radioimmunoassay

Ang II levels were measured by iodine-125 radioimmunoassay (RIA) using the Ang II^125^I radioimmunoassay kit (Buhlmann Laboratories, Switzerland) according to the manufacturer's instructions [41]. Briefly, cells were homogenized in 0.5 ml of saline containing angiotensinase inhibitor (0.1 ml of bestatin solution, Buhlmann Laboratories, Switzerland). The homogenate was centrifuged at 12,000g for 20 min at 4°C. The Ang II concentration was measured in the supernatant with a 1470 Automatic Gamma Counter (Perkin Elmer, Shelton, CT) and calculated with a standard curve generated for each experiment.

### Electrophoretic mobility shift assay (EMSA)

Cultured cells were collected and nuclear proteins were prepared using a Nuclear Protein Extraction kit (Beyotime Institute of Biotechnology). The double-stranded oligonucleotides (Beyotime Institute of Biotechnology) containing the consensus sequence for NF-κB (5′-AG TTGAGGGGACTTTCCCAGGC-3′) were end-labeled with biotin and used as probes for the EMSA. Nuclear extracts (10 μg) were incubated with the binding buffer (10 mM Tris-HCl, pH 7.5, 50 mM NaCl, 0.5 mM dithiothreitol, 0.5 mM EDTA, 2.5% (v/v) glycerol, and 1 μg/μl poly dI-dC) for 10 min and then mixed with 0.5 pmol-labeled probes for 20 min at room temperature. The DNA-protein complex was separated on a 6% native polyacrylamide gel in 0.5× Tris/borate/EDTA buffer at 4°C and transferred onto positively charged nitrocellulose membranes (GE Healthcare Bio-Sciences). The competition assay was performed by adding 100-fold excess of an unlabeled oligonucleotide as a specific competitor. Bands were detected using the SuperSignal™ West Pico Chemiluminescent Substrate, as described by the manufacturer [42].

### Statistical analysis

All experiments were performed three times and the data was expressed as mean ± SD. Statistical analyses were performed by one-way ANOVA using SPSS 17.0 and GraphPad Prism 5.0 software. A *P* value < 0.05 was considered to be statistically significant.

## SUPPLEMENTARY MATERIALS FIGURES AND TABLES


