# Supplementary material for: Effect of captopril on radiation-induced TGF-β1 secretion in EA.Hy926 human umbilical vein endothelial cells
Source: Oncotarget. 2017 Feb 15;8(13):20842–50. doi: 10.18632/oncotarget.15356 (PMC5400550; doi:10.18632/oncotarget.15356)

Effect of captopril on radiation-induced TGF-β1 secretion in EA.Hy926 human umbilical vein endothelial cells

Supplementary Materials

After 14 × 2 Gy radiations, we found that in the process of ionizing radiation, radiation-sensitive EA.Hy926 cells showed elongated cell proliferation cycle (Reproductive cycle elongated from 3 days into 7 days.), slow growth rate, smaller and synapses shorter cells and circular direction growth pattern.

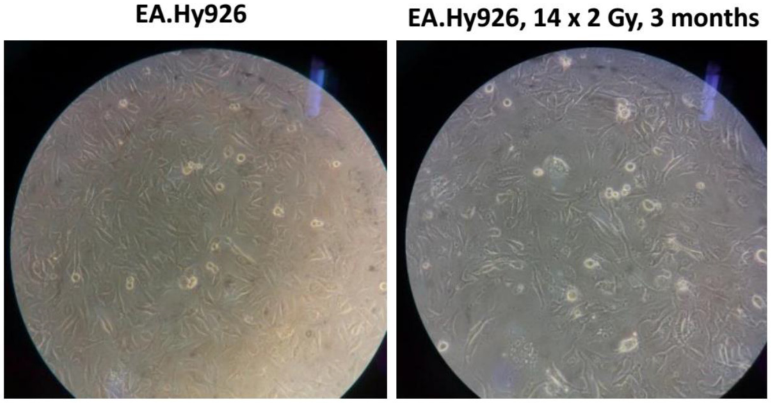

Supplement: Supplementary file 1 [file oncotarget-08-20842-s001.pdf]
